# Dynorphin Activates Quorum Sensing Quinolone Signaling in Pseudomonas aeruginosa

**DOI:** 10.1371/journal.ppat.0030035

**Published:** 2007-03-16

**Authors:** Olga Zaborina, Francois Lepine, Gaoping Xiao, Vesta Valuckaite, Yimei Chen, Terry Li, Mae Ciancio, Alex Zaborin, Elaine Petroff, Jerrold R Turner, Laurence G Rahme, Eugene Chang, John C Alverdy

**Affiliations:** 1 Department of Surgery, Pritzker School of Medicine, University of Chicago, Chicago, Illinois, United States of America; 2 Institut National de la Recherche Scientifique (INRS)–Institut Armand-Frappier, Laval, Quebec, Canada; 3 Department of Surgery, Harvard Medical School, Massachusetts General Hospital, Boston, Massachusetts, United States of America; 4 Department of Medicine, Pritzker School of Medicine, University of Chicago, Chicago, Illinois, United States of America; 5 Department of Biochemistry and Molecular Biology, Pritzker School of Medicine, University of Chicago, Chicago, Illinois, United States of America; 6 Department of Immunohistochemistry, Pritzker School of Medicine, University of Chicago, Chicago, Illinois, United States of America; 7 Department of Pathology, Pritzker School of Medicine, University of Chicago, Chicago, Illinois, United States of America; University of California San Diego, United States of America

## Abstract

There is now substantial evidence that compounds released during host stress directly activate the virulence of certain opportunistic pathogens. Here, we considered that endogenous opioids might function as such compounds, given that they are among the first signals to be released at multiple tissue sites during host stress. We tested the ability of various opioid compounds to enhance the virulence of Pseudomonas aeruginosa using pyocyanin production as a biological readout, and demonstrated enhanced virulence when P. aeruginosa was exposed to synthetic (U-50,488) and endogenous (dynorphin) κ-agonists. Using various mutants and reporter strains of *P. aeruginosa,* we identified involvement of key elements of the quorum sensing circuitry such as the global transcriptional regulator MvfR and the quorum sensing-related quinolone signaling molecules PQS, HHQ, and HQNO that respond to κ-opioids. The in vivo significance of κ-opioid signaling of P. aeruginosa was demonstrated in mice by showing that dynorphin is released from the intestinal mucosa following ischemia/reperfusion injury, activates quinolone signaling in *P. aeruginosa,* and enhances the virulence of P. aeruginosa against *Lactobacillus* spp*.* and *Caenorhabditis elegans.* Taken together, these data demonstrate that P. aeruginosa can intercept opioid compounds released during host stress and integrate them into core elements of quorum sensing circuitry leading to enhanced virulence.

## Author Summary

Precisely how bacterial pathogens such as Pseudomonas aeruginosa cause fatal infections in critically ill humans is unknown. Evidence suggests that a major source of infection may be the patient's own intestinal microflora, which is subjected to unusual environmental conditions during critical illness. Here, we show that intestinal P. aeruginosa can be alerted to the presence of a physiological disturbance in its host by dynorphin, a human morphine-like chemical released during severe stress. Exposure of P. aeruginosa to dynorphin activates its virulence machinery to produce harmful toxins and to suppress the growth of probiotic bacteria, which are known to promote intestinal health. The molecular mechanisms of these events involve the activation of highly regulated virulence machinery in *Pseudomonas,* called quorum sensing, that allows bacteria to sense host stress and respond with enhanced harmfulness. These observations suggest that opportunistic pathogens like P. aeruginosa are equipped with sophisticated surveillance systems that take advantage of a weakened host by intercepting and responding to naturally occurring host chemicals that are normally used as signaling molecules for immune activation and analgesia. Elucidation of the effect of dynorphin on *Pseudomonas* exposes a major mechanism by which this organism behaves as a true opportunist.

## Introduction

It has been suggested for microbial pathogens that colonize the mucosal surface of a healthy host that symbiosis can be viewed as a form of molecular détente, a settled negotiation that is sustained by an ongoing chemical dialogue between the host and its flora [1]. Even for an opportunistic pathogen, virulence expression against its host presents a fundamental tradeoff in that it will provoke immune retaliation and deplete the host of critical resources, and, as such, bacteria are constantly assessing the costs versus benefits of expressing virulence. Although bacteria use complex systems of communication like the quorum sensing signaling (QS) system to collect, process, and share information about the chemical composition of their environment [2], whether such events are influenced by specific host-derived signals that indicate a major change in host health status is less well defined.

Our laboratory has been interested in host-derived bacterial signaling compounds that are proximate causes of microbial virulence activation during physiologic stress. To date, several host-derived bacterial signaling compounds have been identified that include adaptive elements of the immune system such as interferon γ [3], tumor necrosis factor α [4], and interleukin-1 [5], as well as innate elements including adenosine [6], epinephrine [7–10], and antimicrobial peptides [11–13]. While in vitro exposure to various host compounds can activate the virulence of bacteria, much remains to be learned about how these compounds are collected, processed, and transduced within the various virulence regulatory systems of bacteria.

One of the best studied systems of virulence regulation in bacteria is the QS system. The QS system functions via autoinducer molecules that are released and taken up by bacteria to provide a cell–cell communication network whereby complex assemblage behavior can be carried out by large populations of bacteria responding to local concentrations of QS molecules [2]. In some cases, host-derived bacterial signaling molecules such as epinephrine have been shown to act as a surrogate QS autoinducer molecule [8], activating various virulence genes in intestinal bacteria such as Escherichia coli. In other cases, the QS system is activated by the binding of host-derived bacterial signaling molecules to specific membrane receptors on the bacteria, such as when interferon γ binds to the OprF outer membrane protein in Pseudomonas aeruginosa [3].

We considered that opioids might function as host-derived bacterial signaling molecules given that endogenous opioids are broadly distributed within the richly innervated intestinal mucosa and exert multiple effects during stress in neuronal, immune, and intestinal epithelial cells [14–17]. The intestinal tract represents a unique intersection of opioids and bacteria given the high abundance of peripheral neurons, immune cells, and bacteria in this site. Three main families of opioids have been identified based on their affinity to δ-, μ-, and κ-opioid receptors [14] that include the endogenous opioids β-endorphin, enkephalin, and dynorphin [18–20]. Following stress, endogenous opioids have been shown to act as paracrine and autocrine signals with high levels of functional redundancy and pleiotropy [21]. These observations, coupled with the findings that neutrophils themselves can synthesize and release opioids at sites of inflammation, strongly suggest that bacteria are exposed to opioids during the course of infection.

We have been interested in the mechanism by which the human opportunistic pathogen P. aeruginosa is activated to express a virulent phenotype during stress. We have previously shown in mice that stress results in the release of soluble compounds into the intestinal lumen that directly activate the virulence of P. aeruginosa to disrupt the intestinal epithelial barrier [22]. Given the abundance of neurons and immune cells in the gut that could produce opioids, we exposed strains of P. aeruginosa to various opioids with specificity to μ-, λ-, and κ-opioid receptors and used pyocyanin (PCN) production as a biologic readout for virulence expression. Results demonstrated that only the κ-opioid receptor agonist U-50,488 induced PCN production in a dose-dependent manner. Next, we examined the effect of dynorphin, a naturally occurring κ-opioid peptide known to be present in the mammalian intestine, on its ability to produce PCN in *P. aeruginosa,* and found that dynorphin potently induced PCN production. In an in vivo stress model in mice, we demonstrated that dynorphin is released into the intestinal lumen and binds to desquamated epithelia and intestinal *P. aeruginosa.* Dynorphin was found to penetrate the bacterial membrane and directly induce the expression of the multiple virulence factor regulator (MvfR)–regulated operon *pqsABCDE,* resulting in enhanced production of three known intercellular QS-related signals, 2-heptyl-4-hydroxyquinoline N-oxide (HQNO), 4-hydroxy-2-heptylquinoline (HHQ), and *Pseudomonas* quinolone signal (3,4-dihydroxy-2-heptylquinoline [PQS]) [23]. Exposure of P. aeruginosa strain PAO1 to κ-opioid receptor agonists U-50,488 and dynorphin resulted in enhanced virulence as judged by suppressed growth of the probiotic microorganisms Lactobacillus spp. and the nematode Caenorhabditis elegans. Taken together, these studies provide novel insight into the mechanism by which P. aeruginosa is activated to express virulence in response to host stress by processing the opioid peptide dynorphin into its QS circuitry.

## Results

### U-50,488 Stimulates P. aeruginosa PAO1 to Produce Pyocyanin

Preliminary work in our laboratory demonstrated that exposure of P. aeruginosa to filtered intestinal contents from stressed mice induced an intensely green color (unpublished observation), suggesting that the blue-green pigment PCN, a known quorum sensing–dependent virulence factor [24], was produced by soluble compounds released into the intestinal tract during stress. In order to determine whether opioid compounds might be among the factors responsible for bacterial virulence activation during stress, we exposed P. aeruginosa PAO1 to μ-, κ-, and δ-opioid receptor agonists, and examined bacteria for a change in color and PCN production. Studies were performed using morphine, a predominately μ-opioid agonist previously shown to be synthesized in animals [25,26], U-50,488, a specific synthetic κ-opioid, and BW373U86, a specific synthetic δ-opioid agonist [14]. Figure 1A shows that κ-opioid U-50,488 induced an intensely bright green color in P. aeruginosa PAO1 that correlated to an increase in PCN production in a dose-dependent manner (Figure 1B). The δ-opioid BW373U86, on the other hand, had an inhibitory effect on PCN production (Figure 1C), whereas exposure to the μ-agonist morphine resulted in a bell-shaped type dose response curve for PCN (Figure 1D). Although exposure to morphine did not increase PCN to the same degree as with U-50,488 (∼40% versus 300%), at lower cell densities when baseline levels of PCN were negligible, the effect of morphine appeared to be pronounced at the 50-μM dose (∼600%) (Figure 1E). None of the opioids tested resulted in significant changes in the growth of P. aeruginosa (Figure 1F–1H).

**Figure 1:**
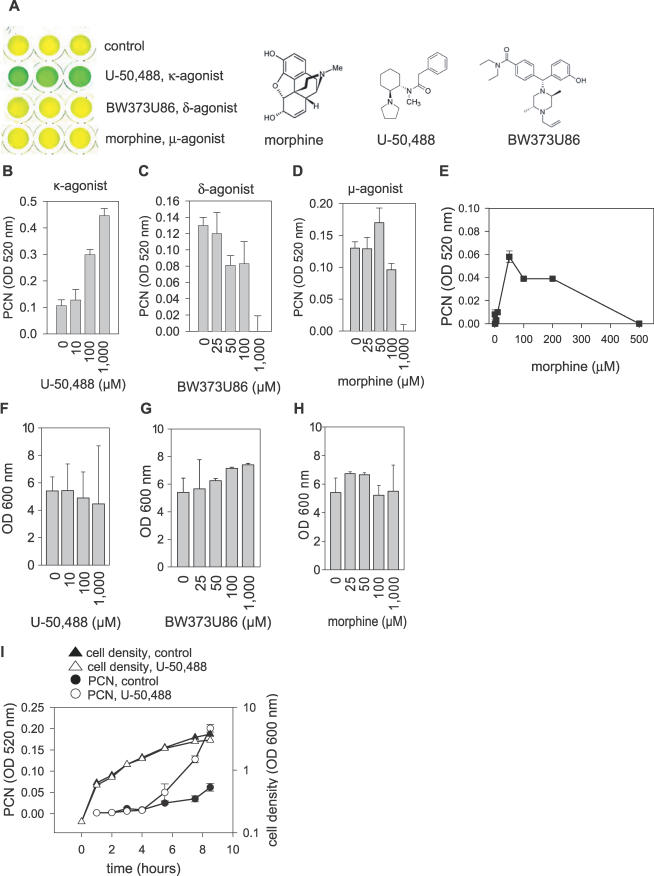
U-50,488 Induces P. aeruginosa PAO1 to Produce PCN Error bars are mean ± SD. (A) Changes in cell culture color in PAO1 following overnight exposure to 1 mM of κ- (U-50,488), δ- (BW373U86), and μ- (morphine) opioid receptor agonists. (B–D) Production of PCN in response to (B) κ-agonist U-50,488, (C) δ-agonist BW373U86, and (D) μ-agonist morphine. (E) Dose response curve of PCN production in PAO1 exposed to morphine. (F–H) Effect of opioids on growth of P. aeruginosa PAO1. (I) Dynamic tracking of PCN production in PAO1 exposed to 200 μM U-50,488.

Since PCN production is a quorum sensing–dependent virulence factor and is produced at high bacterial cell densities, we analyzed the effect of U-50,488 during bacterial growth. U-50,488 induced PCN production at earlier cell density without affecting bacterial growth, suggesting a regulatory shift in the quorum sensing circuitry of P. aeruginosa (Figure 1I).

### Production of PCN in Response to U-50,488 Requires an Intact QS System: Role of Proximal QS Regulatory Protein MvfR in Enhanced PCN Production in Response to U-50,488

Figure 2A outlines the critical pathways within the QS system that are involved in the regulation of PCN production. To verify that κ-opioid mediated activation of PCN production requires an intact QS system, P. aeruginosa PAO1 mutants defective in key transcriptional regulators RhlR, LasR, GacA, and MvfR, as well as the autoinducer synthetases RhlI and LasI, were exposed to U-50,488 at concentrations of up to 1 mM. All transcriptional regulator mutants failed to produce PCN both in the presence and absence of U-50,488, suggesting that an intact QS system is necessary for the κ-opioid–mediated effect (unpublished data). PCN production was partially restored in ΔRhlI and ΔLasI mutants by adding exogenous C4-HSL (Figure 2B). That the addition of C4-HSL had a minimal effect in the RhlI mutant can be explained by competitive binding of 3-oxo-C12-HSL to RhlR in the absence of C4-HSL. In the double mutant ΔLasIΔRhlI, PCN production was increased in response to C4-HSL, possibly as a result of the absence 3-oxo-C12-HS. In contrast to C4-HSL, the κ-opioid agonist U-50,488 failed to restore PCN production under the same conditions, suggesting that κ-agonists cannot function as surrogate QS molecules. We next focused on two key proximal QS regulatory proteins, MvfR and GacA, which are known to be critically important in PCN biosynthesis. The mutants were complemented with their respective genes, *mvfR* and *gacA*. While both complemented mutants produced PCN, responsiveness to the κ-agonist U-50,488 was only observed in ΔMvfR/*mvfR* (Figure 2C). Dynamic tracking of PCN production in the ΔMvfR/*mvfR* during growth again demonstrated enhanced PCN production in response to the κ-agonist (Figure 2D). An inhibitory effect of U-50,488 on PCN production was found in the complemented ΔGacA mutant (Figure 2C). The mechanism for this paradoxical effect is unknown. It is possible that complex interactions between the GacA and MvfR regulons that develop in the presence of high copies of GacA in the complemented mutant and in MvfR when activated by U-50,488 produce this dampening effect.

**Figure 2:**
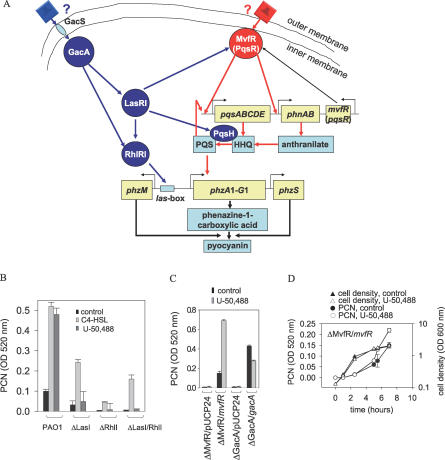
Role of Proximal QS Regulatory Protein MvfR in Enhanced PCN Production in Response to U-50,488 Error bars, mean ± SD. (A) Schematic of PCN regulation in P. aeruginosa. (B) PCN production in ΔRhlI and ΔLasI mutants exposed to exogenous C4-HSL, 1 mM and U-50,488, 1 mM. (C) Production of PCN in ΔMvfR complemented with *mvfR* (ΔMvfR/*mvfR*) and ΔGacA complemented with *gacA* (ΔGacA/*gacA*) genes on a pUPC24 plasmid, or transformed with blank plasmid (ΔMvfR/pUCP24, ΔGacA/pUCP24) in the absence (control) or presence of U-50,488, 1 mM. (D) Dynamic tracking of PCN production in complemented mutant ΔMvfR/*mvfR* grown in the presence of 200 μM U-50,488.

### Exposure of P. aeruginosa PAO1 to U-50,488 Results in Enhanced Expression of *pqsABCDE,* Production of HQNO, HHQ, and PQS, and PA-I Lectin Expression

Because MvfR directs the transcription of the *pqsABCDE* operon [27], which is responsible for the biosynthesis of 2-heptyl-4-hydroxyquinoline N-oxide (HQNO) as well as the direct precursor of the *Pseudomonas* quinolone signal HHQ [23], we exposed PAO1 to U-50,488 and examined the effect of U-50,488 on the expression of *pqsABCDE* and the production of HQNO, HHQ, and PQS. The expression of *pqsABCDE* was examined by measuring β-galactosidase activity in strain PAO1 harboring the pGX5 plasmid containing *pqsA'-lacZ* construction [28]. Figure 3A shows that exposure of PAO1 to U-50,488 resulted in enhanced expression of *pqsABCDE*. Next, we examined the effect of U-50,488 on *mvfR* expression in strain PAO1 harboring the pGX1 containing the *mvfR'-lacZ* fusion gene [29] and found that U-50,488 had no effect on *mvfR* expression (Figure 3B). The concentrations of PQS, HHQ, and HQNO were found to be elevated in PAO1 exposed to U-50,488 (Figure 3C). No differences were observed in the production of other important QS molecules C4-HSL and 3-oxo-C12-HSL in PAO1 grown in the presence or absence of U-50,488 (unpublished data). It has been recently reported that exposure of P. aeruginosa to PQS significantly increases PA-I lectin (PA-IL) expression [30]. Since U-50,488 enhanced PQS biosynthesis, we considered it might also stimulate PA-IL expression. PA-IL expression was dynamically tracked in response to U-50,488 using the green fluorescent PA-IL reporter strain 27853/PLL-EGFP previously constructed in our laboratory [31]. Marked differences in fluorescence were observed in this strain during growth in the absence and presence of U-50,488 (Figure 3D). Results were confirmed in strain PAO1 by real-time PCR (Figure 3E), demonstrating the increased expression of the *lecA* gene encoding PA-IL following exposure to U-50,488. Expression of the housekeeping gene *gltA* encoding citrate synthase was analyzed under the same conditions, and no effect of U-50,488 on *gltA* expression was observed.

**Figure 3:**
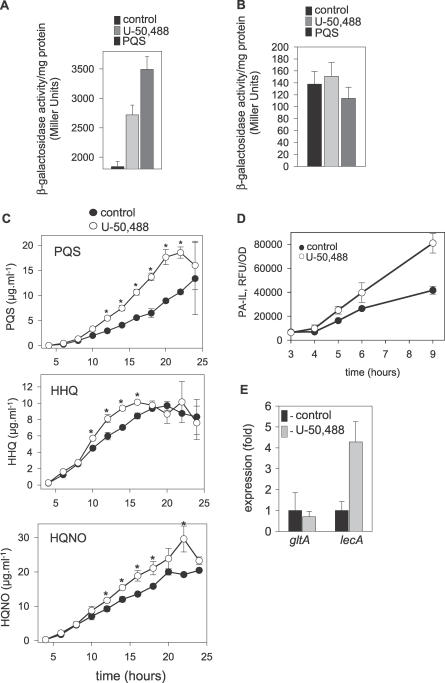
U-50,488 Induces *pqsABCDE* Expression, Biosynthesis of HQNO, HHQ, and PQS, and Stimulates PA-IL Expression Error bars, mean ± SD. (A) Effect of U-50,488, 200 μM and PQS, 100 μM on *pqsA'-lacZ* expression in P. aeruginosa strain PAO1/pGX5 following 5 h of incubation. (B) Effect of U-50,488, 200 μM and PQS, 100 μM on *mvfR'-lacZ* expression in P. aeruginosa strain PAO1/pGX1 following 5 h of incubation. (C) Effect of U-50,488, 200 μM on HQNO, HHQ, and PQS production by P. aeruginosa PAO1. * *p* < 0.01. (D) Dynamic tracking of PA-IL expression using PA-IL reporter strain P. aeruginosa 27853/PLL-EGFP. (E) Real-time PCR of *lecA* encoding PA-IL and the housekeeping gene *gltA* encoding citrate synthase in P. aeruginosa PAO1 grown to OD_600nm_ = 3.0 in the presence of 200 μM of U-50,488. The graph was made based on the Ct levels for *gltA,* 20.26±0.81 (control) versus 20.78±0.26 (U-50,488); and for *lecA,* 29.53±0.43 (control) versus 27.42±0.97 (U-50,488). Ct levels for *lecA* blank control (no template) were ∼ 40.

### The Naturally Occurring κ-Opioid Peptide Dynorphin Enhances the Expression of *pqsABCDE,* Leading to Increased Production of HQNO, HHQ, and PQS, the Expression of *phzA1-G1,* and Enhanced Biosynthesis of PCN

Having established that opioid-induced PCN production in P. aeruginosa is specific to κ-receptor agonists, we next sought to determine whether naturally occurring endogenous κ-agonists could induce PCN production in P. aeruginosa. Among endogenous opioids, only dynorphin has been shown to be specific to the κ-receptor [32]. Therefore, we exposed PAO1 to varying concentrations of dynorphin A (1–17) (Sigma) and found a dose-dependent effect of dynorphin on PCN production (Figure 4A). We next determined if dynorphin increased the expression of *mvfR, pqsABCDE,* and *phzA1*-*G1,* key components involved in PCN regulation. The fusion constructs *mvfR'-lacZ* on pGX1 [23], *pqsA'-lacZ* on pGX5 [28], and *phzABC-lacZ* on MW303 [33] were introduced into strain PAO1. Similar to U-50,488, dynorphin did not increase MvfR expression in PAO1/*mvfR'-lacZ* (unpublished data); however, dynorphin increased β-galactosidase activity in both PAO1/*phzABS-lacZ* (Figure 4B) and PAO1/*pqsA'-lacZ* (Figure 4C). When PAO1/*pqsA'-lacZ* was exposed to both dynorphin and PQS, β-galactosidase activity was increased above that observed with either dynorphin or PQS alone (Figure 4D), suggesting a synergistic effect of dynorphin and PQS on *pqsABCDE* expression.

**Figure 4:**
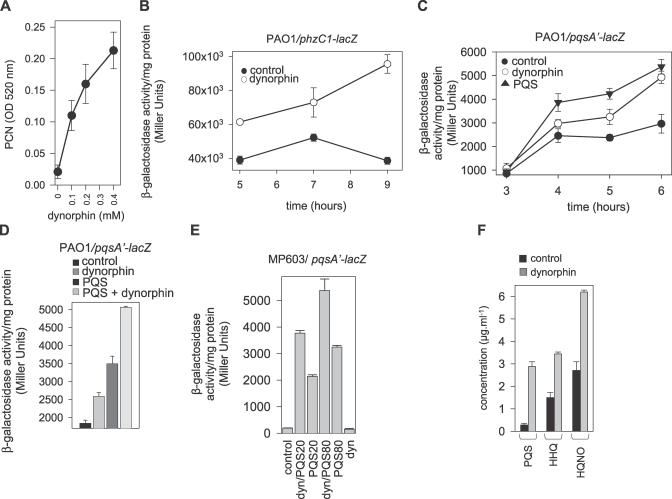
Dynorphin Activates MvfR-Dependent Pathway in P. aeruginosa PAO1 Error bars, mean ± SD. (A) Dose-dependent effect of dynorphin on PCN production. (B) Expression of *phzC1-lacZ* in PAO1/pMW303 in the absence (control) or presence of 100 μM of dynorphin. (C) Dynamic tracking of expression of *pqsA'-lacZ* in PAO1/pGX5 grown in the presence of dynorphin, 100 μM, or PQS, 100 μM. (D) Expression of *pqsA'-lacZ* in PAO1/pGX5 in response to dynorphin, 100 μM, or PQS, 100 μM, or dynorphin plus PQS (100 μM each) determined after 5 h of incubation. (E) Expression of *pqsA'-lacZ* in MP603/pGX5 in response to dynorphin, 100 μM, or PQS (20 and 80 μM), or sum of dynorphin (100 μM) and PQS (20 μM); or sum of dynorphin (100 μM) and PQS (80 μM) determined after 5 h of incubation. (F) Concentration of HQNO, HHQ, and PQS in P. aeruginosa PAO1 after 8 h of growth in the absence (control) or presence of dynorphin, 100 μM.

Next, we determined if dynorphin increased *pqsABCDE* expression in the absence of PQS. We used a PAO1 derivative *pqsC* knockout mutant, strain MP603 [34]. Strain MP603/*pqsA'-lacZ* displayed only a baseline level of β-galactosidase activity both in the presence and absence of dynorphin; however, β-galactosidase activity was significantly increased in response to the combination of dynorphin and PQS compared to PQS alone (Figure 4E). The synergistic effect on *pqsABCDE* expression in strain MP603 was dependent on the relative concentrations of dynorphin and PQS. For example, no synergy was observed when the PQS concentration exceeded that of dynorphin, and similarly, an inhibitory effect was observed when the dynorphin concentration (>10-fold) exceeded that of PQS (unpublished data). Finally, similar to U-50,488, dynorphin increased HQNO, HHQ, and PQS production in PAO1 (Figure 4F).

### Dynorphin Accumulates in Intestinal Tissues during Stress, Is Released into the Intestinal Lumen, and Is Transferred to Bacteria within the Intestinal Lumen

We hypothesized that bacteria might be exposed to dynorphin in vivo in the intestinal tract under clinically relevant pathophysiological conditions [35]. To test this, we exposed the mouse intestine to two conditions: 1) 30 min of ischemia followed by 30 min of reperfusion stress, and 2) ischemia/reperfusion (I/R) stress coupled with luminal inoculation with P. aeruginosa (I/R + Pa). Figure 5A–5C shows 4-μm intestinal sections isolated from (A) control, (B) I/R, and (C) I/R + Pa mice, and stained with hematoxylin and eosin. The black arrows on Figure 5B and 5C show desquamated epithelium that is a common feature of this injury. Figure 5D–5G shows immunohistochemical staining of intestinal segments for dynorphin. In control samples (Figure 5D and 5G), dynorphin was found to be scarcely localized to the crypts (Figure 5D, red arrow), whereas following I/R injury, dynorphin was found to be abundantly present on the villus tips and within the intestinal lumen (Figure 5E and 5H, red arrows), a finding that appeared to be enhanced in the presence of luminal P. aeruginosa (Figure 5F and 5I, red arrows). Examination of bacteria within the intestinal lumen and on the epithelial surface demonstrated positive dynorphin staining bacteria at various sites, including bacteria attached to intestinal epithelial cells (Figure 5J–5M).

**Figure 5:**
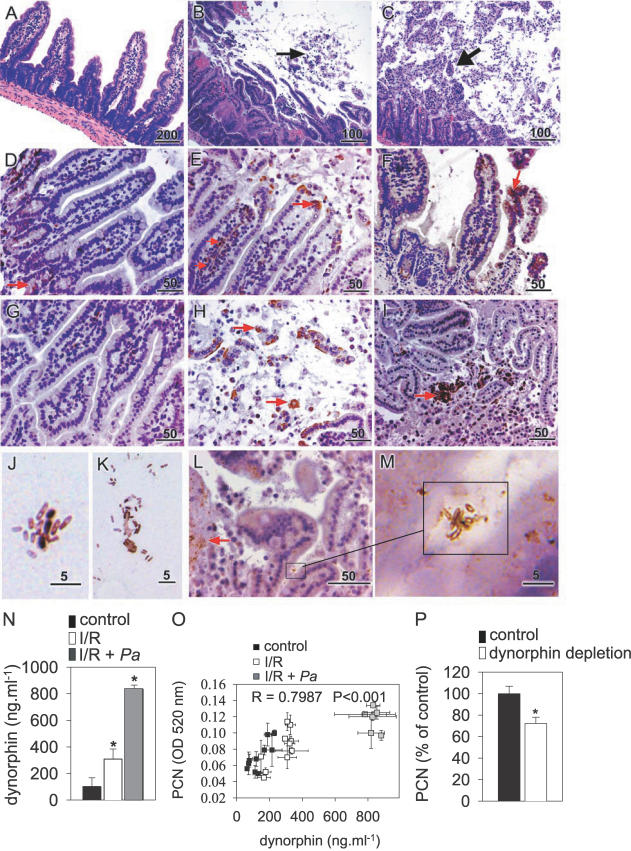
In Vivo Production of Dynorphin in the Mouse Intestine during I/R (A–C) Histology of small intestine from (A) control mice demonstrating intact mucosal epithelium, and (B) I/R and (C) I/R + Pa mice showing disruption of mucosal epithelium with desquamated epithelial cells inside the intestinal lumen (black arrows). (D–F, G–I) Immunohistochemistry of the small intestine from (D and G) control mice demonstrating scarce dynorphin localized to the epithelial crypt (brown staining, red arrow), and following (E and H) I/R and (F and I) I/R + Pa showing dynorphin migration up the lamina propria (red arrows, [E]) and its accumulation on villi and within the lumen. Scale bars are in μm. (J–M) Images of luminal bacteria from mouse small intestine subjected to I/R + Pa demonstrating (J) transfer of dynorphin to bacteria (brown-colored bacteria) and (K) positive dynorphin stained bacteria bound to desquamated epithelia; (I) abundant epithelial dynorphin staining (red arrow), and (M) co-localization of dynorphin stained luminal bacteria to sites of dynorphin accumulation at the epithelial surface. Scale bars are in μm. (N**)** Concentration of dynorphin in filtered luminal flushes isolated from intestine of control mice and mice subjected to I/R and I/R + Pa. *n* = 10/group, * *p* <0.001. (O) Correlation analysis between dynorphin concentration in luminal flushes and their ability to induce PCN production in PAO1. (P) Effect of dynorphin depletion with anti-dynorphin antibody on the ability of luminal flush samples to produce PCN in PAO1, *n* = 6/group, * *p* <0.005. Error bars, mean ± SD.

To determine the concentration of dynorphin in the luminal contents of intestinal segments subjected to I/R and I/R + Pa, 10-cm segments were flushed with 2 ml of phosphate buffered saline (PBS) containing protease inhibitor cocktail (Roche), and samples assayed using competitive enzyme-linked immunosorbent assay (ELISA). Figure 5N shows a significant increase in luminal dynorphin in mice subjected to I/R injury that was further increased when I/R was coupled with luminal inoculation with P. aeruginosa (I/R + Pa). To define the putative role of dynorphin on PCN production in vivo, PAO1 was exposed to filtered (0.22 μm) luminal contents from each group of mice, and PCN production determined. Exposure of PAO1 to luminal flushings from intestinal segments of the various groups of mice demonstrated a significant correlation between dynorphin concentration in the luminal samples and its ability to induce PCN production (R = 0.7987, Figure 5O). Immunodepletion of dynorphin in samples using rabbit polyclonal anti-dynorphin antibody attenuated the ability of samples to induce PCN production in PAO1 (Figure 5P).

### Dynorphin Binds to P. aeruginosa In Vitro and Enters the Bacterial Cytoplasm

To confirm that dynorphin can bind to bacteria, we performed in vitro staining of P. aeruginosa in the presence of dynorphin. Dynorphin (100 μM) was added to P. aeruginosa at the early log phase, and incubated for 1 h. Cells were collected, washed, and fixed on slide. Dynorphin was detected by immunostaining using anti-dynorphin pAB. Figures 6A and 6B show negative dynorphin staining in the negative controls when cells were cultivated without dynorphin (A), and when cells were cultivated with dynorphin but primary antibodies were omitted and rabbit serum was used instead (B). Figure 6C demonstrates positive dynorphin staining in cells cultivated with dynorphin followed by treatment with anti-dynorphin antibody. Structurally, dynorphin is similar to other cell-penetrating peptides in that its high content of basic and hydrophobic amino acid residues facilitates its penetration through mammalian cell membranes [36]. Therefore, by using immunogold electron microscopy, we determined the ability of dynorphin to traverse the bacterial plasma membrane and enter the bacterial cell interior. Figure 6D shows an image of P. aeruginosa PAO1 cells incubated with dynorphin. Localization of dynorphin was identified by 10-nm gold particles (black arrows), which were found predominantly in the bacterial cytosol fraction close to the inner membrane.

**Figure 6:**
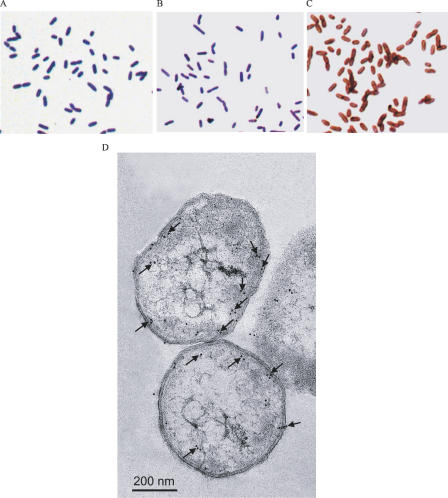
Dynorphin Binds to P. aeruginosa In Vitro, and Enters the Bacterial Cell Cytoplasm (A–C) Binding of dynorphin to *P. aeruginosa;* (A) negative control demonstrating no dynorphin staining when cells were not incubated with dynorphin; (B) negative control demonstrating no dynorphin staining when cell were incubated with dynorphin but primary anti-dynorphin antibodies were omitted from staining procedure; and (C) positive staining (brown color) of P. aeruginosa incubated with dynorphin followed by whole procedure of immunostaining. (D) Immunoelectron microscopy of P. aeruginosa PAO1 cells incubated with dynorphin, 100 μM. Black arrows show 10-nm gold spots indicating the presence of dynorphin.

### κ-Opioid Receptor Agonists U-50,488 and Dynorphin Enhance the Virulence of P. aeruginosa PAO1 against *Lactobacillus* and C. elegans

To determine the clinical relevance of the above findings, we examined the ability of κ-opioid receptor agonists to shift the virulence of P. aeruginosa against the nematode C. elegans. In addition, we determined whether exposure P. aeruginosa to κ-opioid receptor agonists could affect the growth of the cytoprotective probiotic organisms Lactobacillus plantarum and Lactobacillus rhamnosus [37–40]. Media from P. aeruginosa PAO1 grown in the presence of U-50,488 suppressed the growth of L. plantarum and L. rhamnosus GG (Figure 7A and 7B), whereas U-50,488 alone had no effect (unpublished data). Similarly, media from P. aeruginosa PAO1 grown in the presence of dynorphin suppressed the growth of *Lactobacillus,* whereas dynorphin alone had no effect (Figure 7C and 7D). Conditioned media from the PAO1 ΔMvfR mutant grown in the presence or absence of dynorphin did not affect the growth of Lactobacillus spp., suggesting that the κ-mediated effect is regulated via MvfR (Figure 7E). C. elegans feeding on lawns of PAO1 exposed to U-50,488 or dynorphin demonstrated suppressed production of new progeny, an indicator of enhanced virulence (Figure 7F and 7G). In this assay, the PAO1 ΔMvfR mutant was observed to be significantly less virulent compared to the wild-type PAO1 (Figure 7F), and its virulence was not enhanced in the presence of κ-agonists (Figure 7F and 7G).

**Figure 7:**
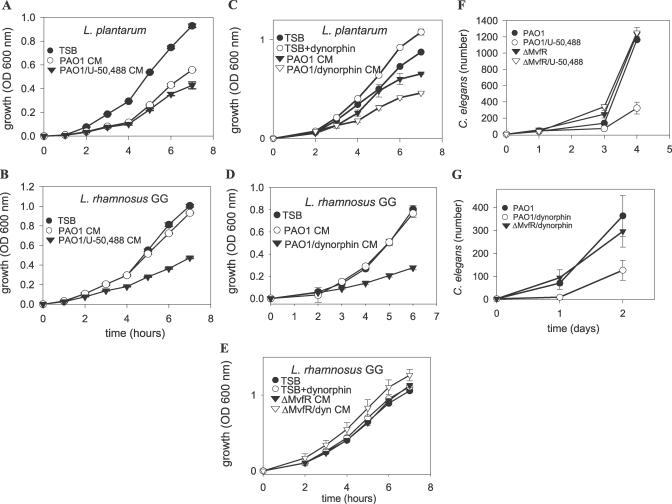
κ-Opioid Receptor Agonists Activate Virulence of P. aeruginosa against Probiotic Bacteria and C. elegans Error bars, mean ± SD. (A and B) The exposure of P. aeruginosa PAO1 to U-50,488, 200 μM, increases the inhibiting effect of its extracellular milieu (conditioned media) on the growth of probiotic microorganisms (A) L. plantarum and (B) L. rhamnosus GG. (C and D) The exposure of P. aeruginosa PAO1 to dynorphin, 100 μM, increases the inhibiting effect of its extracellular milieu (conditioned media) on the growth of probiotic microorganisms (C) L. plantarum and (D) L. rhamnosus GG. (E) The extracellular milieu of P. aeruginosa PAO1 mutant ΔMvfR exposed to dynorphin, 100 μM, did not inhibit the growth of probiotic microorganism L. rhamnosus GG. (F and G) P. aeruginosa PAO1 but not mutant ΔMvfR exposed to (F) U-50,488, 200 μM, or (G) dynorphin, 100 μM, suppressed the production of new progeny in C. elegans.

## Discussion

In animals exposed to physiologic or traumatic stress, subsequent bacterial challenge has been shown to result in increased mortality [10,31] in association with impaired immune function and bacterial clearance [17,41,42]. Data from the present study add to the small but expanding body of data showing that soluble compounds released by the host during stress and immune activation can directly interact with pathways of bacterial virulence regulation in a highly specific manner [3,8,11]. Opioids are ubiquitous neurotransmitters within the enteric nervous system and encompass a wide variety of functions, including motility, secretion, immune modulation, and maintenance of epithelial barrier function. The abundance of the neural network within the intestinal tract is matched only by its microbial flora, where bacterial cells outnumber the total number of cells in the body [14]. In this study, we found κ-opioid receptor agonists to induce PCN production in P. aeruginosa. Although dynorphin has been shown to be present in a variety of tissues, whether dynorphin accumulates in intestinal tissues following host stress and/or bacterial infection has not been previously addressed. Data from the present study show for the first time to our knowledge, that dynorphin is released into the intestinal lumen following I/R injury and accumulates in regions of high dynorphin concentration, such as on desquamated epithelium, where it binds to P. aeruginosa. Binding of dynorphin to P. aeruginosa was further confirmed in vitro by direct antibody staining. In addition, the penetration of dynorphin into the cytosolic compartment of bacterial cells was demonstrated by immunoelectron microscopy. These findings, coupled with the observation that dynorphin can activate the virulence of P. aeruginosa, may be of significant clinical relevance given that intestinal ischemia invariably accompanies physiologic stress and has been associated with fatal infection due to intestinal P. aeruginosa [43,44]. However, dynorphin may enhance the virulence of P. aeruginosa not only within the intestine but also at other sites of tissue injury and inflammation. There is overwhelming evidence that opioids are released and accumulate at sites of inflammation primarily because all of these tissues sites are heavily innervated and highly populated by macrophages and neutrophils. [45,46]. The ubiquitous presence of opioid receptors on nerves throughout the body suggests that exposure of P. aeruginosa to opioids may be one of the reasons it has evolved a mechanism to respond to these compounds [45,46]. Therefore, P. aeruginosa virulence could be activated by dynorphin in all infections associated with inflammation, including burns, implanted medical devices, and lung infections in patients with underlying lung disease.

The precise mechanisms by which colonizing pathogens important to human disease process host signals for the purposes of virulence activation is a small and poorly understood area of investigation. The pathways by which bacteria gather, process, and become activated by host signals have been shown to be both specific to bacteria and specific to the host signal involved, and gene activation through the QS system has been previously reported [3,8]. A major mechanism by which the κ-opioid receptor agonists U-50,488 and dynorphin affect virulence gene activation in P. aeruginosa could be via expression of the *pqsABCDE* operon. The *pqsABCDE* operon directs the biosynthesis of 4-hydroxy-2-alkylquinolines (HAQs), among which HHQ and PQS are themselves signaling molecules. Additionally, HHQ is a direct precursor of PQS [23]. PQS functions as a regulatory link between the LasRI and RhlRI quorum sensing systems [30,47], and has been shown to play a critical role in the pathogenesis of P. aeruginosa in nematodes, plants, and mice [24,48,49]. Data from the present study suggest that dynorphin synergizes with PQS to increase *pqsABCDE* expression. Further experiments are underway to clarify the precise mechanism of dynorphin activation of *pqsABCDE* expression.

Among critically ill and immunocompromised patients, infection with P. aeruginosa carries the highest case fatality rate of all nosocomial pathogens, approaching 60% [50,51]. The primary site of colonization for P. aeruginosa in such patients is the gastrointestinal tract, where as many as 50% of hospitalized patients harbor this organism [52,53]. Risk factors for mortality due to P. aeruginosa infection suggest that the degree of host stress is a major determinant of a fatal outcome from this pathogen [53,54]. In the present work we show (Figure 8) that P. aeruginosa presents a “triple threat” to its host when exposed to dynorphin in that it can 1) activate QS circuits via enhanced PQS production, 2) reduce populations of protective probiotic bacteria such as lactobacilli through the production of HQNO and PCN [55], and 3) increase the production of QS-dependent virulence gene products that affect host cell function such as PCN and the PA-I lectin/adhesin [30,56–58]. A clearer understanding of the mechanisms by which P. aeruginosa is activated to express virulence in direct response to host stress has the potential to lead to preventive therapies that interdict in the process of infection at its most proximate point.

**Figure 8:**
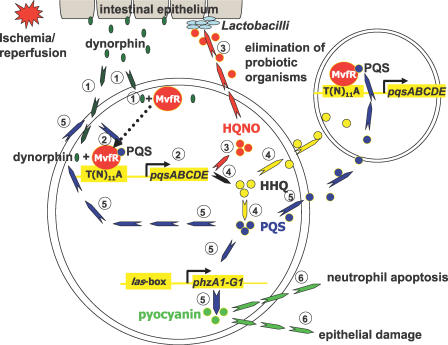
Proposed Activation and Effectors Pathways of P. aeruginosa in Response to Host Stress (Intestinal I/R Injury) (1) Dynorphin is released by intestinal tissues and accumulates in the lumen during ischemia/reperfusion and penetrates the plasma membrane of P. aeruginosa (dark green arrows). (2) Dynorphin synergizes with PQS via MvfR to increase the transcription of *pqsABCDE* leading to the production of HAQs, including HQNO and HHQ. (3) Increased HQNO production suppresses the growth of Lactobacillius spp., rendering the intestinal epithelium more vulnerable to invasion and the action of cytotoxins of P. aeruginosa (red arrows). (4) HHQ is the immediate precursor of PQS [23], and both compounds play an important role in bacterial cell-to-cell communication [23] (yellow and blue arrows). (5) PQS induces the expression of *pqsABCDE* [63], and is required for *phzA1-G1*expression*,* the gene responsible for PCN production (blue arrows). 6.) The release of PCN can induce neutrophils apoptosis and damage epithelial cells [64] (green arrows) allowing for immuno-evasion and deeper penetration of bacteria.

## Materials and Methods

### Bacterial strains and culture conditions.

P. aeruginosa strain PAO1 and its derivatives were routinely grown in tryptic soy broth (TSB) supplemented when necessary with tetracycline (Tc), 60 μg/ml, gentamicin (Gm), 100 μg/ml, or carbenicillin (Cb), 300 μg/ml. P. aeruginosa strains PAO1 wild-type, ΔRhlR (*rhlR::* ISphoA/hah, ID 44488), and ΔMvfR (*mvfR::* ISlacZ/hah, ID 13375) were obtained from the P. aeruginosa mutant library [59]. Strains PAO-R1 (ΔLasR (*lasR*::Tc), PAO-JP-1 (ΔLasI (*lasI*::Tc), and PDO100 (ΔRhlI (*rhlI*::Tn*501*) were kindly provided by B. Iglewski. Strains PAO6281 (ΔGacA (*gacA*::Spr /Sm), PAO-MW1 (ΔRhlIΔLasI (*rhlI*::Tn*501 lasI*::*tetA*), and MP603 (*pqsC*::Tc) were kindly provided by C. Reimmann, P. Greenberg, and C. Manoil, respectively.

### PCN assay.

P. aeruginosa cultures were grown at 37 °C, under shaking conditions at 220 rpm, in TSB supplemented with either morphine (Abbott Laboratories, http://www.abbott.com), U-50,488 (Sigma, http://www.sigmaaldrich.com), or BW373U86 (Sigma) at varying concentrations. Following overnight incubation, bacterial cells were spun down by centrifugation at 10,000*g* for 5 min, and 1 ml of supernatant was extracted using 500 μl of chloroform, re-extracted with 150 μl of 0.2 M HCl, and then PCN was measured at OD 520 nm as described [60]. In experiments with dynorphin A (1–17) (Sigma), 200 μl of bacterial culture was incubated in 2-ml wells of a 96-well microplate (Whatman, http://www.whatman.com). Following overnight incubation, 200 μl of chloroform and 150 μl of 0.2 M HCl were used to extract PCN from cell-free culture media. In dynamic experiments, PAO1 was grown in TSB supplemented with 200 μM U-50,488, and 1-ml aliquots were serially collected. Cell density was measured at OD 600 nm, and samples were processed for PCN assay. In experiments in which strains complemented with *mvfR* or *gacA* on the pUCP24 plasmid were used, experiments were performed with a higher dose of U-50,488 (1 mM), as a baseline of PCN production was already high due to these strains containing multi-copy plasmids encoding MvfR or GacA.

### The β-galactosidase assay.

Plasmid pGX5 [28] harboring the *pqsA'-lacZ* fusion construction was introduced in P. aeruginosa strains PAO1 and MP603; and plasmids pGX1 harboring the *mvfR'-lacZ* fusion [23] and pMW303 harboring the *phzABC-lacZ* fusion [33] were introduced in PAO1 by electroporation. Single colonies of PAO1/pGX5 or PAO1/pGX1, or PAO1/pMW303 were used to inoculate TSB supplemented with carbenicillin (Cb), 300 μg/ml, for overnight growth at 37 °C, 220 rpm. Overnight culture was diluted 1:100 in TSB, and 300 μg/ml Cb was supplemented with U-50,488, 200 μM, or dynorphin, 100 μM, or PQS, 100 μM, or without supplementation (control). Aliquots of cells cultures were taken out dynamically; pellet was collected by centrifugation at 6,000*g,* 5 min, and kept at −80°C before processing. MP603/pGX5 was inoculated in TSB supplemented with Tc, 60 μg/ml and Cb, 300 μg/ml. Overnight cultures were diluted 1:100 in TSB, subgrown for 1 h to a cell density of OD_600nm_ 0.07–0.1, and aliquoted to create the following groups (in triplicates): 1) “control”, 2) “dynorphin”, dynorphin added to a final concentration of 100 μM; 3) “PQS”, PQS added to final concentrations of 20 and 80 μM; and 4) “dynorphin + PQS”, dynorphin added to a final concentration of 100 μM, incubated for 1 h, followed by the addition of PQS to a final concentration of 20 and 80 μM. All cell cultures were collected at the same time (5 h), cell density of 1:10 dilutes samples was measured at OD 600 nm, and cells were collected by centrifugation at 6,000*g,* 5 min, and kept at −80°C before processing. For β-galactosidase activity analysis, cells were treated with protein extraction reagent (BugBuster Master Mix; Novagen, http://www.emdbiosciences.com/html/NVG/home.html) for 20 min at room temperature, centrifuged for 30 min at 4 °C, and cytosol fractions were used to measure β-galactosidase activity. In this case, 15 μl of cytosol fractions were loaded on 96-well plates, followed by 135 μl of a master mixture of o-nitrophenyl-β-D-galactopyranoside (ONPG, Sigma) containing 100 μl of 0.1 M Na-phosphate buffer (pH 7.5), 33 μl of ONPG, 4 mg/ml dissolved in 0.1 M Na-phosphate buffer (pH 7.5), and 2 μl of Mg buffer (63 μl of H2O, 35 μl of 2-mercaptoethanol, and 2 μl of 5 M MgCl_2_). Absorbance at 410 nm was measured using Plate Reader, and results were expressed as Miller units (1,000 × Δ410 nm × mg protein^−1^ × min^−1^).

### RNA isolation and cDNA synthesis.

For RNA isolation, 1 ml of P. aeruginosa PAO1 culture was grown in TSB with or without 200 μM U-50,488, to OD_600 nm_ = 3.0. Next, 2 ml of RNA Protect Bacteria reagent (Qiagen, http://www1.qiagen.com) was added immediately at the end of the incubation period, and samples treated as recommended by the Qiagen's lysis protocol, followed by the addition of 3 ml of TRIzol LS reagent (Invitrogen, http://www.invitrogen.com). The RNA enrichment fraction was separated using Phase lock gel, heavy (Eppendorf, http://www.eppendorf.com). RNA was precipitated with isopropanol, dissolved in water, and the remaining DNA degraded using DNA-Free kit (Ambion, http://www.ambion.com). RNA integrity was monitored by formaldehyde agarose gel electrophoresis, and the absence of DNA checked by PCR using primers for 16S r RNA, forward 5′-GGACGGGTGAGTAATGCCTA-3′ and reverse 5′-CGTAAGGGCCATGATGACTT-3′. The first-strand cDNA was prepared using 2 μg of total RNA, Superscript II RNase H–RT (Invitrogen), and random primers as recommended by the manufacturer's protocol.

### Real-time reverse transcription (RT)–PCR*.*

Real-time PCR was performed on the ABI Prizm 7300 Sequence Detection System using SYBR Green qPCR SuperMix-UDG (Invitrogen), cDNA, and respective primers: for *gltA* encoding citrate synthase (PA1580), 5′TCTACCACGACTCCCTGGAC3′ and 5′TTTTCCGCGTAGTTCAGGTC3′; for *lecA* encoding PA-IL (PA2570), 5′CGATGTCATTACCATCGTCG3′ and 5′ACCCTGGACATTATTGGGTG3′. The integrity of the RT-PCR products was confirmed by melting-curve analysis. Expression levels were calculated based on differences in Ct levels.

### Protein concentration assay.

Protein was measured using the BCA Protein Assay Reagent (Pierce, http://www.piercenet.com).

### HQNO, HHQ, and PQS quantification.

A single colony of PAO1 was used to inoculate 5 ml of TSB. Overnight culture was diluted in fresh TSB at 1:100, supplemented with either U-50,488 (200 μM) or dynorphin (100 μM). After 20 min of incubation under shaking conditions, cell cultures were aliquoted as 650 μl in 14-ml culture tubes. At designed time points, three tubes from each group were removed, and 650 μl of MeOH containing 2% acetic acid was immediately added, properly mixed, replaced into Eppendorf tubes, centrifuged at 13,000*g,* 30 min, 4 °C, and the supernatant used to quantify HQNO, HHQ, and PQS by HPLC/MS according to Lépine et al. [61]. Unlabeled PQS was obtained by the same synthetic route described for deuterium-labeled PQS [61]. The final PQS-d4 concentration was 20 mg/l, and the stock solutions were in methanol.

### C4-HSL and 3-oxo-C12-HSL quantification.

The homoserine lactones were quantitated by LC/MS/MS using a water/acetonitrile gradient containing 1% acetic acid. The analyses were performed in positive electrospray ionization mode and the acquisitions were obtained in Multiple Reaction Mode (MRM). The transitions monitored were m/z 298 to 102 for the N-(3-oxododecyl)-homoserine lactone and m/z 172 to 102 for the N-(butyroyl)-homoserine lactone. Argon at 2.0 × 10^−3^ mtorr was the collision gas and the collision energy was 15 eV.

### Complementation of MvfR mutant with *mvfR* gene*.*

The *mvfR* gene was amplified using primers forward 5′-AAGGAATAAGGGATGCCTATTCA-3′ and reverse 5′-CTACTCTGGTGCGGCGCGCTGGC-3′ and cloned in pCR2.1 (Invitrogen). Plasmid pCR2.1/*mvfR* was digested with XbaI-HindIII restrictases, and *mvfR* was subcloned in pUCP24 under the *Plac* promoter [62] to create pUCP24/*mvfR*. The plasmids pUCP24 and pUCP24/*mvfR* were electroporated in strain 13375 defective in MvfR production to create strains ΔMvfR/pUCP24 (control) and ΔMvfR/*mvfR* .

### Complementation of GacA mutant with *gacA* gene.

The *gacA* gene was amplified using primers forward 5′-CGACGAGGTGCAGCGTGATTAAGGT-3′ and reverse 5′-CTAGCTGGCGGCATCGACCATGC-3′ and cloned in pCR2.1 (Invitrogen). Plasmid pCR2.1/*gacA* was digested with XbaI-HindIII restrictases, and *gacA* was subcloned in pUCP24 under the *Plac* promoter to create pUCP24/*gacA*. The plasmids pUCP24 and pUCP24/*gacA* were electroporated in strain PAO6281 defective in GacA production to create strains ΔGacA/pUCP24 and ΔGacA/*gacA*.

### Anti-MvfR antibody.

Polyclonal antiserum against 50–63 peptide LVRRDGYKVEPTEQ of PA1003 (MvfR) was produced in rabbits (ZYMED Laboratories, http://www.invitrogen.com). Anti-MvfR antibodies were affinity purified by AminoLink Plus Immobilization Kit (Pierce) using 50–63 peptide to create an affinity column.

### Segmental intestinal I/R model.

All experiments on mice were performed in accordance with University of Chicago guidelines and regulations, and mouse protocol number 71629 was approved by the Animal Care and Use Committee of the University of Chicago. Male, wild-type C57Bl/6 mice (8- to 10–wk-old; Charles River, http://www.criver.com) were fasted overnight prior to use in the I/R studies. For these studies, mice were lightly anesthetized with sevoflurane prior to an i.p. bolus injection of Avertin (Sigma-Aldrich #T4, 840–2) prepared as a 1.2 % solution, at 0.2 ml × 10 g^−1^ body weight. Mice were placed on a warmed heating pad under a warming light. An abdominal midline incision was made and the small intestines exposed. Sutures were placed so as to isolate a 20-cm segment of small intestine. The intestine was divided proximally and distally using cautery. A clamp was placed on the superior mesenteric artery (SMA) to occlude blood flow to the jejunum and ileum. Ischemia was confirmed by visible blanching of the intestinal segment. During the ischemic and reperfusion period, mice were hydrated via i.p. administration of warmed saline. After 30 min of ischemia, the SMA clamp was removed and the intestinal tract allowed to reperfuse for 30 min prior to removal of the isolated segment. This segment was then immediately flushed with 2 ml of PBS containing a protease inhibitor cocktail (Roche, http://www.roche.com). Mice were euthanized by an overdose of anesthesia, followed by cervical dislocation. In selected experiments, P. aeruginosa (Pa) was injected into the isolated intestinal segment prior to SMA clamping at a concentration of 10^7^ CFU/ml in 2 ml of PBS to recapitulate the clinical circumstance of I/R occurring in the presence of an opportunistic infection (I/R + Pa).

### Histological and immunohistochemical staining.

For histology, specimens were fixed in 10% buffered formalin and embedded in paraffin. Paraffin specimens were cut into 4-μm sections and were mounted on micro slides. Hematoxylin and eosin–stained slides were then reviewed using an Olympus microscope. For immunohistochemical staining, sections were deparaffinized and re-hydrated through xylen and serial dilutions of ETOH to distilled water. Section were then incubated in antigen retrieval buffer (DAKO, S1699; http://www.dako.com) and heated in a steamer at 98 °C degree for 20 min. After rinsing, slides were incubated in 3% hydrogen peroxide for 5 min and then 10% normal goat serum in 0.025% Triton X-100-PBS for 30 min. Anti-dynorphin A (1–17) antibody (EMD Biosciences, http://www.emdbiosciences.com) at 1:25 dilution was applied for overnight incubation at 4 °C in a humidity chamber. Following a TBS wash, slides were incubated with secondary antibody (DAKO, K4011) and the antigen–antibody binding was detected by DAB substrate chromogen system (DAKO, K3466). To ensure that positive staining was specific, control experiments were performed as outlined above, eliminating the primary antibodies, and showed no staining. Slides were briefly immersed in hematoxylin for counterstaining and evaluated under Zeiss Axioskop (http://www.zeiss.com), using oil 25× Korr objective na 1.3 and oil 63× apochromat objective na 1.4 with pixel sizes 1280 × 1024 regular and 3840 × 3072 highest.

### In vitro immunostaining of P. aeruginosa PAO1 incubated with dynorphin.

Cells of *P. aeruginosa* were grown overnight in TSB and diluted 1:100 with 400 μl of fresh TSB media. After 2 h of incubation, cells were added by dynorphin, 100 μM (when needed), and incubated for one more hour. Cells were collected, washed three times with PBS, and fixed in 4% paraformaldehyde for 2 h, 4 °C, at rotation. Then, cells were kept on ice for 45 min followed by intensive washing with PBS. After last washing, cells were resuspended in 200 μl of PBS, and 100 μl of cell suspension was poured on slide. The fixation of cells to slide was allowed overnight at room temperature. Cells on slides were then incubated in antigen retrieval buffer (DAKO, K4011) and followed the above procedure of immunostaining (see “Histological and immunohistochemical staining”). Two negative controls were performed: 1) omitting dynorphin, and 2) omitting primary anti-dynorphin antibodies.

### Immunoelectron microscopy*.*

P. aeruginosa PAO1 was grown for 10 h in the presence of dynorphin, 100 μM, and then cells collected by centrifugation, 5,000*g,* 5 min, washed with PBS, and fixed with 4% paraformaldehyde plus 0.1% glutaraldehyde in 0.1 M phosphate buffer (pH 7.4) (PB) for 1 h. After fixation, cells were washed with 0.1 M PB, and gradually dehydrated using ethanol at increasing concentrations from 30% to 100%. Infiltration was performed using 100% ethanol:LR White Medium (Electron Microscopy Sciences http://www.emsdiasum.com/microscopy) (1:1) for 1 h followed by overnight infiltration with LR White Medium. After infiltration, cells were embedded in gelatin capsules. Polymerization was performed in a vacuum oven at 45 °C for 48 h. Then, 80-nm thickness sections were cut using a Reichert-Jung Ultracut E, and mounted on formvar-coated 200-mesh nickel grids. Sections on grids were re-hydrated with PBS for 30 min, blocked with 1% BSA in PBS for 30 min, and primed with rabbit anti-dynorphin A 1–17 antibody (EMD Biosciences) at 1:25 dilution in 1% BSA at humidified chamber for 3.5 h. Grids were extensively washed with PBS, blocked with 0.1% BSA in PBS for 25 min, and incubated in a humidified chamber for 1 h with goat anti-rabbit IgG conjugated with 10-nm gold particles (Ted Pella, http://www.tedpella.com) at 1:10 dilution in 0.1% BSA. Grids were washed with PBS, fixed with 1% glutaraldehyde in PBS for 10 min, washed with water, and stained briefly with uranyl acetate and lead citrate. Air dried grids were examined at 300 kV with an FEI Tecnai F30 (FEI, http://www.fei.com). Two negative controls were used: 1) grids without incubation with rabbit anti-dynorphin A 1–17 antibody, and 2) PAO1 grown in the absence of dynorphin.

### Biotinylation of dynorphin*.*

Dynorphin A (1–17) (Sigma) was biotinylated using NHS-PEO_4_-biotin (Pierce), and purified by HPLC.

### Competitive ELISA for detection dynorphin in luminal flushings*.*

Luminal flushings were filtered with 0.22-μm filters (Millipore, http://www.millipore.com), aliquoted, and stored at −80 °C. Affinity purified F(ab)_2_ of Frag Donk anti-Rb IgG (Jackson Immunological Research Laboratories, http://www.jax.org) at a concentration of 10 μg/ml in carbonate-bicarbonate buffer (Sigma) were coated onto Maxisorp immunomodules (Nunc, http://www.nuncbrand.com) for 2 h at 37 °C. Unbound sites were blocked with 3% bovine serum albumin in PBS for 30 min at room temperature. After blocking, rabbit anti-dynorphin A (1–17) antibodies (EMD Biosciences) at 1:250 dilution in PBST (PBS, Tween 0.05%) were added to wells for 1 h, at 37 °C. After washing with PBST, mixtures of biotinylated dynorphin, 50 ng/ml with varying concentrations of unlabeled dynorphin (0, 20, 50, 100 ng/ml) or filtered luminal flushings diluted in buffer (20 mM phosphate buffer (pH 7.4), 150 mM NaCl, 10 mM EDTA, 0.5% BSA, 0.02% Triton X-100) were loaded for 2 h at 37 °C. In this manner biotinylated dynorphin competes for antibody binding sites with unlabeled dynorphin or dynorphin in luminal flushes. After incubation, unbound biotinylated peptide was removed by washing with PBST, and ImmunoPure streptavidin-conjugated horseradish peroxidase (Pierce) was added and allowed to bind to the immobilized primary antibody-biotinylated peptide complex. After washing, O-phenylaminediamine (Sigma) was allowed to react with the bound HRP. The color intensity that develops is dependent on the quantity of biotinylated peptide bound to the immobilized antibody. When more non-biotinylated peptide competes for the limited amount of antibody, less biotinylated peptide/SA-HRP can be immobilized and less color is produced by the substrate

### PCN assay in PAO1 induced by luminal flushings.

PAO1 was exposed to 20 μl of 0.22 μm-filtered luminal flushings included in 2 ml of TSB, and PCN was measured after overnight growth. For immunodepletion, samples were pre-incubated with anti-dynorphin antibody (EMD Biosciences) at 1:100 dilution for 2 h.

### Effect of conditioned media from P. aeruginosa on the growth of Lactobacillus spp.

Overnight cultures of P. aeruginosa PAO1 were used to inoculate fresh TSB (1:100, vol/vol). After inoculation, the PAO1 culture was subgrown for 1 h, then aliquoted, and U-50,488 or dynorphin were added to aliquots to create final concentrations of 200 μM and 100 μM, respectively. After 23 h of growth at 37 °C, 180 rpm, cultures were collected, centrifuged (5,000 rpm, 5 min) to remove cells, and supernatant was filtered using Millex-CV low protein binding membrane filters of 0.22-μm pore size (Millipore). The filtered supernatant, now termed conditioned media, was stored on ice before use. L. plantarum and L. rhamnosus GG grown overnight in MRS broth (Oxoid, http://www.oxoid.com) at 37 °C were used to inoculate fresh MRS (2:100, vol/vol). Freshly inoculated culture, 100 μl was put into 96-well plates (Nunc), followed by an addition of 100 μl of conditioned media from P. aeruginosa. Control samples were created where 100 μl of TSB; TSB+U-50,488, 200 μM; TSB+dynorphin, 100 μM were added to wells. Plates with Lactobacillus spp. were incubated at 37 °C, unshaken, and growth was monitored dynamically by measuring OD 600 nm using Plate Reader.

### Virulence assay of P. aeruginosa PAO1 using C. elegans.

Wild-type N2 C. elegans were kindly provided by M. Glotzer, University of Chicago. Culturing, cleaning, egg preparation for synchronization, and transferring were performed according to “Maintenance of C. elegans” (http://www.wormbook.org/chapters/www_strainmaintain/strainmaintain.html). For the experiment, two adult nematodes were transferred to a lawn of P. aeruginosa on NGM agar prepared by the following: P. aeruginosa PAO1 and PAO1 derivative ΔMvfR mutant were grown for 6 h at 37 °C in TSB (control culture) or TSB supplemented with U-50,488 (U-50,488 culture), 200 μM, or TSB supplemented with dynorphin, 100 μM (dynorphin culture). Then, 10 μl of TSB media (control), or TSB supplemented with U-50,488, 1 mM, or dynorphin, 200 μM, was dropped at the center of NGM agar plate, allowed to dry for 10 min, and then 10 μl of bacterial culture was dropped on a respective plate (control culture on TSB spot, U-50,488 culture on U-50,488 spot, and dynorphin culture on dynorphin spot). The plates were incubated at 37 °C overnight and allowed to equilibrate to 20 °C for two h before being seeded with nematodes. The plates with nematodes on P. aeruginosa lawn were incubated for up to four days at 20 °C, and 4 × 10 μl of TSB, or TSB/U-50,488, 1 mM, or TSB/dynorphin, 200 μM, were dropped peripherally at the lawn ring, daily. Total numbers of nematodes were counted daily.

### Data analysis*.*

Statistical analysis of the data was performed using Student's *t*-test. Regression analysis was performed using Sigma plot software.

## Supporting Information

### Accession Numbers

The Entrez Protein (http://www.ncbi.nlm.nih.gov/entrez/query.fcgi?db=Protein) accession numbers for the P. aeruginosa PAO1 gene products discussed in this paper are citrate synthase (NP_250271), GacA (NP_251276), LasI (NP_250123), LasR (NP_250121), MvfR (NP_249694), PA-I galactophilic lectin (NP_251260), RhlI (NP_252166), and RhlR (NP_252167). The Entrez Nucleotide (http://www.ncbi.nlm.nih.gov/entrez/query.fcgi?db=Nucleotide) accession number for the P. aeruginosa PAO1 complete genome is NC_002516.

## Abbreviations

HHQ: 4-hydroxy-2-heptylquinoline
HQNO: 2-heptyl-4-hydroxyquinoline N-oxide
I/R: ischemia/reperfusion
MvfR: multiple virulence factor regulator
PA-IL: PA-I lectin
PBS: phosphate buffered saline
PCN: pyocyanin
PQS: 3,4-dihydroxy-2-heptylquinoline
QS: quorum sensing signaling
