# Correction: Dynorphin Activates Quorum Sensing Quinolone Signaling in Pseudomonas aeruginosa

**DOI:** 10.1371/journal.ppat.0030067

**Published:** 2007-05-25

**Authors:** Olga Zaborina, Francois Lepine, Gaoping Xiao, Vesta Valuckaite, Yimei Chen, Terry Li, Mae Ciancio, Alex Zaborin, Elaine O Petrof, Jerrold R Turner, Laurence G Rahme, Eugene Chang, John C Alverdy

In *PLoS Pathogens,* vol 3, issue 3: doi: 10.1371/journal.ppat.0030035

One of the authors, Dr. Elaine O. Petrof, was incorrectly listed as Elaine Petroff.

